# Supplementary figures and images for: Cytotoxic CD4+ T-cells specific for EBV capsid antigen BORF1 are maintained in long-term latently infected healthy donors
Source: PLoS Pathog. 2021 Dec 9;17(12):e1010137. doi: 10.1371/journal.ppat.1010137 (PMC8691624; doi:10.1371/journal.ppat.1010137)

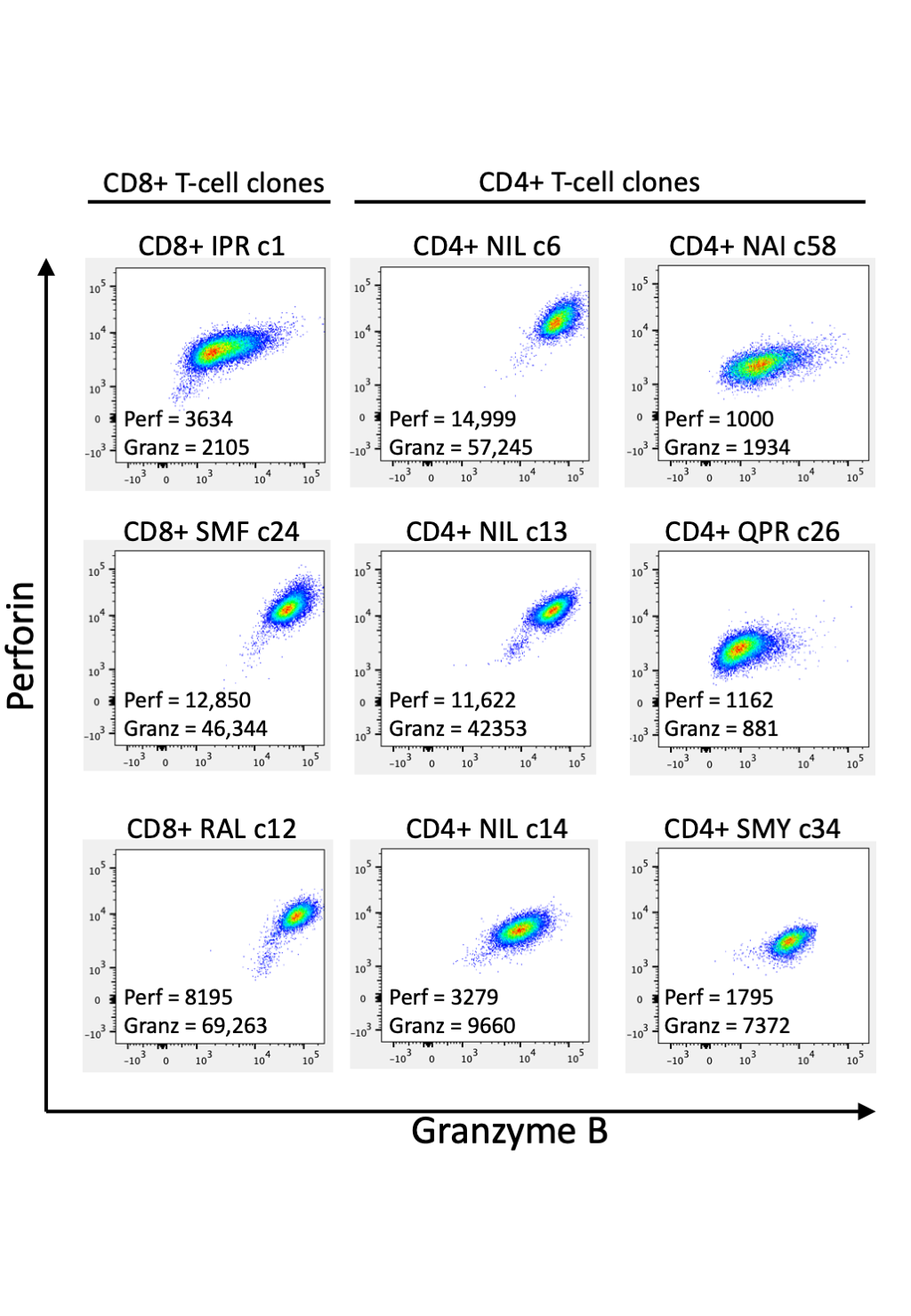

Supplement: S2 Fig — Selected examples of perforin and granzyme B expression in CD8+ (SMF and RAL) and CD4+ (NIL and SMY) capsid specific clones, as measured by flow cytometry. Median Fluorescence Intensity (MFI) values are inset. (TIFF) [file ppat.1010137.s002.tiff]

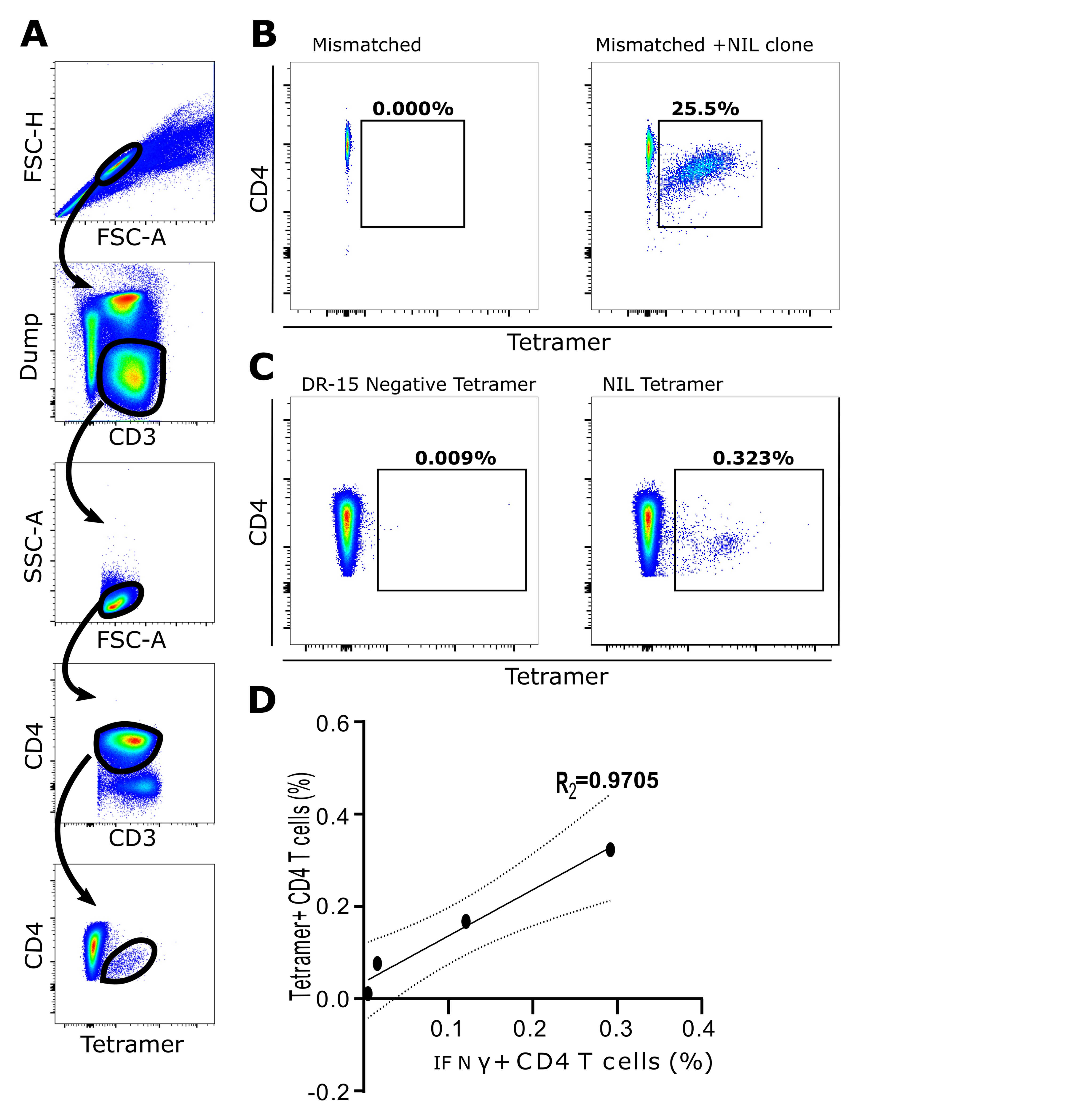

Supplement: S3 Fig — A) Example of the gating strategy used for identification of tetramer positive CD4+ T-cells. First, doublets were excluded, followed by selection of T-cells (CD3+) with exclusion of CD14+, CD19+, viability dye+ cells (Dump). Lymphocytes, based on SSC vs FSC, and subsequently CD3+CD4+ T-cells were then gated before final tetramer-positive cell identification. B) Tetramer was tested using irrelevant T-cell line unspiked or spiked with T-cells from a NIL-specific T-cell clone (spiked at a 1:4 ratio of NIL clone to irrelevant T-cells) to test tetramer specificity, shown are T-cell clones stained with the BORF1 NIL tetramer. C) Tetramer was then tested on PBMC from a donor with a known NIL-specific response alongside an irrelevant tetramer, which was used to set gates. Shown is an example of BORF1 NIL tetramer staining. D) Linear correlation of the percentage of BORF NIL tetramer stained CD4+ T-cells and the frequency of CD4+ T-cells responding to stimulation with NIL peptide in the same individuals using PBMC from 4 donors. (TIF) [file ppat.1010137.s003.tif]

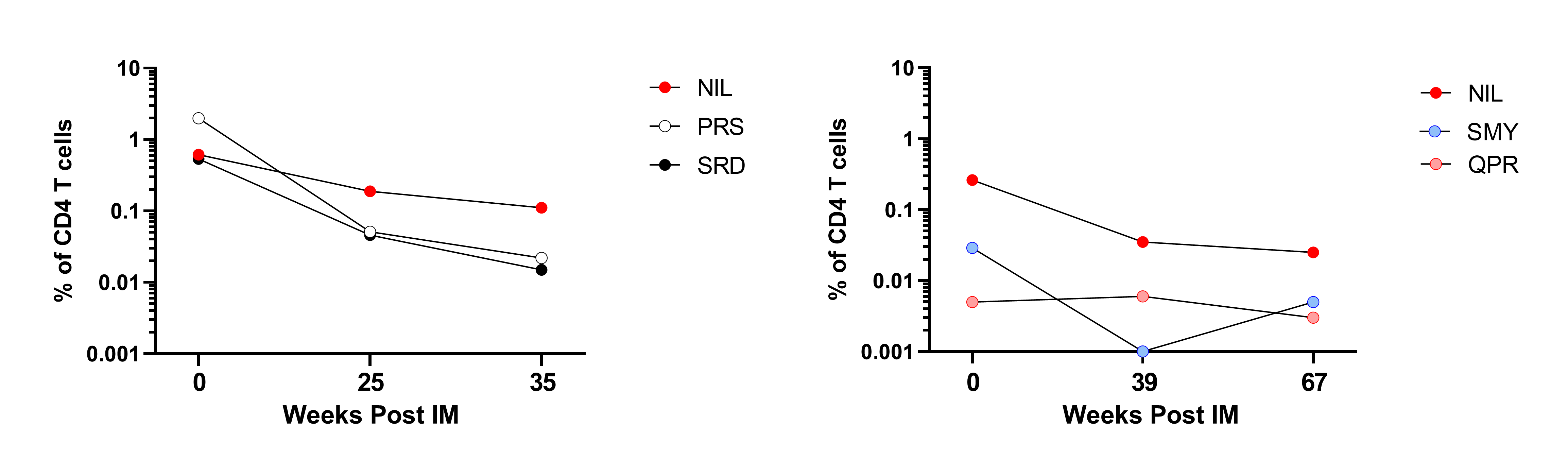

Supplement: S4 Fig — Shown are the frequency of NIL (red) and other tetramer (as indicated) positive CD4+ T-cells during IM (Week 0) and into convalescence (second and third timepoint). Time is relative to the first sample. (TIF) [file ppat.1010137.s004.tif]
