# Supplementary material for: Cytotoxic CD4+ T-cells specific for EBV capsid antigen BORF1 are maintained in long-term latently infected healthy donors
Source: PLoS Pathog. 2021 Dec 9;17(12):e1010137. doi: 10.1371/journal.ppat.1010137 (PMC8691624; doi:10.1371/journal.ppat.1010137)
Supplement: S1 Table — Analysis of VDJ gene usage was performed using the international immunogenetics information system V-Quest tool (http://www.imgt.org). (DOCX) [file ppat.1010137.s005.docx]

| **Clone** | **TCR V-beta sequence** | **V-name** | **D-name** | **J-name** |
| --- | --- | --- | --- | --- |
| 2 | CASSPFPGGPSSYNEQFF | TRBV18*01 | TRBD2*01 | TRBJ2-1*01 |
| 14 | CASSPFPGGPSSYNEQFF | TRBV18*01 | TRBD2*01 | TRBJ2-1*01 |
| 6 | CASSSFPTASMNTEAFF | TRBV18*01 | TRBD1*01 | TRBJ1-1*01 |
| 13 | CASSSFPTASMNTEAFF | TRBV18*01 | TRBD1*01 | TRBJ1-1*01 |
| 3 | CASSPLPNEKLFF | TRBV18*01 | - | TRBJ1-4*01 |
| 5 | CASSPLPNEKLFF | TRBV18*01 | - | TRBJ1-4*01 |
