# Supplementary material for: Cytotoxic CD4+ T-cells specific for EBV capsid antigen BORF1 are maintained in long-term latently infected healthy donors
Source: PLoS Pathog. 2021 Dec 9;17(12):e1010137. doi: 10.1371/journal.ppat.1010137 (PMC8691624; doi:10.1371/journal.ppat.1010137)
Supplement: Attachment — Submitted filename: Dowell et al Rebuttal.pdf [file ppat.1010137.s006.pdf]

Dear Editor,

We thank the reviewers for their careful appraisal of our manuscript. As they noted, identifying new T-cell epitopes that elicit cytotoxic T-cell responses able to recognise newly EBV infected cells is important. We have performed all the additional experiments requested by reviewer three. We have also undertaken substantial alteration of the introduction and discussion text, as requested, to provide greater context for these data. We provide below details of the changes we have made to the manuscript.

Major points

Reviewer 1 asked for additional information on which of the range of EBV-specific T-cell responses may be important in controlling EBV and whether effector function of T-cell clones can be predicted. In response, we have provided additional text in the discussion on the importance of cytolytic CD4+ T-cells against EBV structural antigens and suggest a mechanism by which such T-cells may experience greater levels of stimulation in long-term virus carriers. Regarding a potential link between cytokine production level and cytotoxicity for T-cell clones, given the well characterised skewing of phenotype that occurs during in vitro culture of T-cells we decided not to explore this area instead focusing on the function of the T-cells ex vivo which is more physiological and relevant to vaccine development.

Reviewer 2 provided a detailed critique of the introduction and discussion sections of the manuscript and we thank them for the care they took in doing so. In addition to the changes made in response to reviewer 1 we have added additional text on the biological significance of our results and have also revised the introduction. We now provided T-cell clone restriction using molecular subtypes and have made many additional changes in response to the minor points they made (described below).

Reviewer 3 suggested our data does not show if cytotoxicity is a common feature of T-cells specific for BORF1 epitope NIL, and that the use of T-cell clones provides no insight into the diversity of the T-cell response to NIL or if cytotoxicity is a common feature to all NIL-specific clones. We entirely agree with this limitation of T-cell clones which is exactly why, in the original manuscript, we examined the phenotype of this T-cell response directly *ex vivo* with

MHC-II tetramers and degranulation assays (Figures 5, 6 & 7). These data, clearly more biologically relevant than using T-cell clones, showed, in multiple donors, that NIL-specific CD4+ T-cells express perforin and granzyme and BORF1-specific CD4+ T- degranulate in response to antigen challenge. Both properties are consistent with cytotoxicity.

The reviewer then requested we measure perforin and granzyme-B in the BORF1-specific T-cell clones. We did not do this in the original manuscript because another limitation of T-cell clones is that their phenotype is altered by *in vitro* culture and may differ from the phenotype of the T-cell response *in vivo*. This is why we characterised the response directly *ex vivo*. Nevertheless, we have performed the experiment they requested and provide the results in Figure 4C with example staining in SI Figure 2. The reviewer also raised the question of whether the strong recognition and cytotoxicity exhibited by the NIL-specific T-cells was a feature of a particular T-cell receptor clonotype. Sequencing the TCRs of six clones, we show they possess three different T-cell receptors (SI Table 1) demonstrating that robust recognition of newly infected B-cells is not limited to a single clonotype.

Minor points

Reviewer 1 – no comments made by reviewer

Reviewer 2 – We have revised the introduction and discussion sections in response to these comments and now cite almost all of the papers they suggest (e.g. PMID:16549597, 14581546 and 12133989). We have also included relevant earlier work such as PMID: 8553567. We have not cited PMID:16549599, which was mentioned by the reviewer, as this is an opinion article on one of the primary research papers that is now included (PMID:16549597). In the discussion we have provided a potential explanation for why some epitopes are processed from newly infected B-cells but not LCLs (differences in endosomal protease composition) with appropriate references. We have provided details of the standard assays used to characterise all T-cell clones including their HLA restriction (SI Fig 1). Regarding potential alloreactivity, we are experienced in identifying when this occurs (PMID:16849448) and take precautions to guard against it, such as using LCL lines from the autologous donor (when available) or multiple HLA-matched LCLs when not. Examples shown in the paper include testing T cell clones against multiple partial-matched LCL (SI Fig 1), and the use of control mis-

matched B-cells (e.g. Fig 4B&D). We have made additional minor changes to the text to correct typographical errors identified by the reviewer.

Reviewer 3 – Regarding Figure 3D, while preparing the manuscript we considered both approaches (fixing the y-axis or customising it for each graph). Given that the kinetics of IFNγ change over time is more important than the absolute level of IFNγ we chose to customise the y-axis as that produced the clearest figure. Regarding using ICS rather than IFNγ ELISA to measure T-cell activation, the latter has been used successfully to study antigen processing and presentation for many years by ourselves (e.g. Haigh et al. 2008, Mackay et al. PLoS Pathogens 2009, Leung et al. PNAS 2010, Long et al. JI 2011, Brookes et al. PLoS Pathogens 2016) and others (e.g. Adhikary, Mautner et al. JEM 2006, Fiebiger, Mautner et al. 2012, Fonteneau, Munz et al. JI 2016, Gannange, Munz et al. 2019). The assay is reliable, quantitative and entirely suited to the task.
