# Supplementary material for: Cytotoxic CD4+ T-cells specific for EBV capsid antigen BORF1 are maintained in long-term latently infected healthy donors
Source: PLoS Pathog. 2021 Dec 9;17(12):e1010137. doi: 10.1371/journal.ppat.1010137 (PMC8691624; doi:10.1371/journal.ppat.1010137)
Supplement: Attachment — Submitted filename: Dowell et al Rebuttal.docx [file ppat.1010137.s007.docx]

Dear Editor,

We thank the reviewers for their positive response to the changes which we have made. We have now addressed the final point as requested by providing statistical analysis of our results and as such have added a statistical analysis section to the methods section.

We hope that this meets with your approval.
